# Enhancing tuberculosis vaccine development: a deconvolution neural network approach for multi-epitope prediction

**DOI:** 10.1038/s41598-024-59291-1

**Published:** 2024-05-06

**Authors:** Auwalu Saleh Mubarak, Zubaida Said Ameen, Abdurrahman Shuaibu Hassan, Dilber Uzun Ozsahin

**Affiliations:** 1 Operational Research Centre in Healthcare, Near East University, TRNC Mersin 10, Nicosia, 99138 Turkey; 2 Department of Electrical Engineering, Aliko Dangote University of Science and Technology, Wudil, Kano, Nigeria; 3 Department of Biochemistry, Yusuf Maitama Sule University, Kano, Nigeria; 4 Department of Electrical Electronics and Automation Systems Engineering, Kampala International University, Kampala, Uganda; 5 Department of Medical Diagnostic Imaging, College of Health Science, University of Sharjah, Sharjah, UAE; 6 Research Institute for Medical and Health Sciences, University of Sharjah, Sharjah, UAE

**Keywords:** Computational biology and bioinformatics, Drug discovery

## Abstract

Tuberculosis (TB) a disease caused by *Mycobacterium tuberculosis* (Mtb) poses a significant threat to human life, and current BCG vaccinations only provide sporadic protection, therefore there is a need for developing efficient vaccines. Numerous immunoinformatic methods have been utilized previously, here for the first time a deep learning framework based on Deconvolutional Neural Networks (DCNN) and Bidirectional Long Short-Term Memory (DCNN-BiLSTM) was used to predict Mtb Multiepitope vaccine (MtbMEV) subunits against six Mtb H37Rv proteins. The trained model was used to design MEV within a few minutes against TB better than other machine learning models with 99.5% accuracy. The MEV has good antigenicity, and physiochemical properties, and is thermostable, soluble, and hydrophilic. The vaccine's BLAST search ruled out the possibility of autoimmune reactions. The secondary structure analysis revealed 87% coil, 10% beta, and 2% alpha helix, while the tertiary structure was highly upgraded after refinement. Molecular docking with TLR3 and TLR4 receptors showed good binding, indicating high immune reactions. Immune response simulation confirmed the generation of innate and adaptive responses. In-silico cloning revealed the vaccine is highly expressed in *E. coli.* The results can be further experimentally verified using various analyses to establish a candidate vaccine for future clinical trials.

## Introduction

The Mycobacterium tuberculosis (Mtb) bacteria causes the most fatal infectious illness, tuberculosis (TB). The World Health Organisation (WHO) states that TB is a very infectious disease, there were 10.6 million new cases with 1.6 million deaths in 2021^1–3^. Clinical TB therapy has become very rare, most antimicrobial medication combinations are utilized instead. The current treatments for TB include fluoroquinolones paired with second-line injectables, amikacin, kanamycin, and capreomycin, as well as the first-line drugs isoniazid, rifampicin, ethambutol, and pyrazinamide^4^. It is more likely that Mtb may develop drug-resistant mutations because of the prolonged treatment cycle, which is generally nine to twelve months or longer. Due to the advent and rising prevalence of Mtb which is highly drug-resistant to many drugs, chemotherapy has recently become less effective^5^.

The only approved TB vaccine currently BCG (Bacillus Calmette-Guérin) which is injected intradermally has challenges because of variables in genetic variance among demographics, genetic heterogeneity in BCG strains, the impact of non-tuberculous mycobacteria (NTM), and that of some parasite illnesses that cause death and transmission, therefore the overall efficiency of the TB vaccination is unpredictable^6,7^. Due to problems such as BCG-associated fatalities in immune-compromised infants and several adverse events related to BCG administration, only a small number of nations now practice standard BCG vaccination while a smaller number have embraced the targeted vaccination^8^. Unfortunately, BCG only offers protection to babies and is mostly ineffective against adolescents and adults^9^, since according to the WHO, 89% of TB infections in 2021 were in adults. Therefore, the development of a unique and potent anti-TB vaccination is urgently required, especially for adults and adolescents^3^.

Because of improvements in vaccine research, peptide- and DNA-based vaccines have been designed, which provide scalable and quick treatments. Sixteen TB vaccines are presently undergoing phase I, phase II, and phase III clinical studies, some of which are based on viral vectors or live, attenuated Mtb^10^. However, the use of pDNA within DNA vaccines demonstrated the possibility of insertional mutagenesis^11^. The risk of virulence reversal is reduced when patients are immunized with vaccines based on peptide fragments. Peptide vaccines like H4/IC31 are thought to be stable and perhaps powerful TB vaccinations given their efficacy. H4/IC31 had produced a strong immunological response in healthy adults and newborns who had received the BCG vaccine in phase I tests, and it was clinically safe^12^.

Multi-epitope vaccines have attracted a lot of research recently because they have advantages over standard immunizations, including improved immunity and fewer allergies^13^. An epitope is a segment of an antigen’s protein or amino acid sequence. Epitope recognition by B cells and T cells triggers adaptive immune responses, which are vital for battling the virus. Antibodies that aid in the eradication of infections are secreted by B cells that have been stimulated during the humoral response. As part of the cellular response, T lymphocytes bind epitopes on the surface of host cells which are provided by major histocompatibility complex (MHC) molecules finally killing the infected cells. Multi-epitope vaccines that contain both B-cell and T-cell epitopes can concurrently elicit potent humoral and cellular immunological responses, in contrast to single-epitope vaccines^14^. Multiple in silico techniques were utilized to create a potential vaccine that codes for numerous B and T cell epitopes in the Mtb genome, that may be able to stimulate cellular and humoral immunity^15^. However, in-silico vaccine design procedures may not be quick enough to handle rapidly growing viruses due to the laborious merging and evaluation of data. This adds overhead and time, and now only a single prediction target can be achieved by any in silico vaccine design technology for effective vaccine development^16^. For us to quickly determine the top vaccine components for additional development and testing, no method available today is capable of simultaneously making many predictions and thoroughly analysing the outcomes. The well-known BepiPred^17^, NetMHCpan^18^, and NetMHCIIpan^19^ tools, for instance, are solely used for predictions specific to B-cell, peptide binding to MHC class I and peptide binding to MHC class II alleles, respectively. To create a TB multi-epitope kind of vaccine, this study introduces a deep learning framework for integrated predictions of B cell and T cell epitopes. It was feasible to successfully replace the various crucial predictions and in-depth analysis of epitopes using a deep neural network (DNN) architecture.

## Related works

An ideal TB vaccine should be created to target the proteins/pathways responsible for different characteristics in Mtb to effectively elicit immune responses via T cells^20^. Major Histocompatibility Complexes (MHC), which are highly polymorphic in the host, should also be the focus of an efficient vaccine^21^. The adaptability of the vaccine is demanded to a very high standard by these qualities, which is something that a single natural protein is unable to provide. A unique form of vaccine candidate that may solve the aforementioned problems is the multi-epitope vaccine, a protein that has been reconstructed by several overlapping epitopes (peptides)^22^. Due to their ability to facilitate the research and development of vaccines for a variety of illnesses that are rapidly developing, epitopes are essential for scientific and clinical investigations^23^. A potential vaccine against SARS-CoV-2^16,22,24–26^, malaria^27,28^, Ebola virus^29^, dengue virus field^30^, hepatitis B virus field^31^, *Staphylococcus aureus*^32,33^, *Acinetobacter baumannii*^34–36^, and *Helicobacter pylori*^37^ has been developed using a variety of immuno-informatics techniques. Recently, numerous immunoinformatic tools such as^2,15,24,38,39^ have been utilized in the design of TB epitope-based vaccines.

Utilized were eight Mtb-secreted proteins that are necessary for either pathogenesis or expressed in extracellular space. The epitopes of these proteins were examined to develop a whole vaccine. 10 linear B cell epitopes were picked from 166 anticipated ones, followed by the selection of 16 helper T epitopes that can trigger interferon function from 534 anticipated epitopes, and 15 epitopes from 623 cytotoxic T anticipated epitopes. These epitopes were then conjugated with adjuvant and PADRE, and using the proper linkers. Although the proposed vaccine meets the criteria for a good TB vaccine the epitope selection process is laborious and time-consuming^39^. In another study, the verified Rv0101, Rv3343, and Rv0058 TB antigens were used to create a new multiepitope subunit vaccine. Top choices from forecasted CTL, B-cell, and HTL epitopes were taken into consideration for the whole vaccine with the addition of an adjuvant to improve immunogenicity. Bioinformatics software was used to determine the Mtb epitopes that induce cellular and humoral responses in B and T cells. The NetCTL 1.2 web server was used to predict 24 epitopes, demonstrating how time-consuming this procedure may be. The seven predicted CTL epitopes were ultimately chosen from among them. The IEDB website also led to the identification of HTL epitopes, which are strongly binding MHC class II epitopes for HLA-DR, and eight epitopes were selected. The website ABCpred predicted the B cell epitopes, showing how time-consuming the procedure may be^40^. Similarly, 34 CTL epitopes were predicted from four nominated Mtb proteins for the construction of a Mtb epitope-based vaccination using the NetCTL 1.2 website however, only 10 of these predicted CTL epitopes were employed to build the vaccine. The IEDB website for MHC-II and ABCpred predicted four HTL epitopes and four B-cell epitopes accordingly, which were eventually chosen for the final vaccine. Despite having strong antigenicity scores for the four antigens (Rv3804c and Rv2608, as well as Rv0125, and Rv2684), the developed vaccine is highly antigenic. The AllergenFP service and the AllerTOP v.2 services demonstrated that vaccine sequences are believed to be non-allergenic. Immune modelling showed that there was a general rise in the immunological responses that were evoked following repeated exposure to the antigen, which led to the development of B and T-cells. This shows the involvement of different tools in choosing the best epitopes for the vaccine design^15^. Another study developed a multi-epitope-based vaccine against tuberculosis using extracellular vesicles, or exosomes, which are linked to the development of the illness. Extracellular vesicle proteins with experimentally proven HTL and CTL, B-cell epitopes were selected for the vaccine. The top ten predicted antigenic, as well as non-toxic epitopes but without allergenic properties, were chosen for the vaccine design. Using the ABCpred, the top five B-cell epitopes were predicted. A strong humoral and cellular immune response can be elicited by the suggested vaccine candidate thanks to its excellent structural, fascinating physiochemical, and attractive immunological properties, however, the epitope selection process is quite time-consuming^38^. By causing epigenetic changes, pathogens may influence the transcription of host genes, particularly those involved in the immune system. Numerous Mtb proteins have been shown to modify the epigenome of their hosts. Another study used nine proteins to predict epitopes and create an mRNA vaccine against TB. This vaccine was created using a variety of in-silico methods to stimulate both cellular and humoral immunity. Only eight B-cell epitopes were taken from the nine proteins under study, and these were the top five predicted epitopes from the ABCpred online site for each included protein. From the nine proteins, 17 epitopes were also chosen to be incorporated as CTL epitopes, along with a handful of potential HTL epitopes, to create a final vaccine containing 30 epitopes^41^. A vaccine was developed for the treatment and prevention of SARS-CoV-2 and Mycobacterium tuberculosis (Mtb) coinfection. The outer membrane protein A, also known as Rv0899 of Mtb, and the spike glycoprotein of SARS-CoV-2 each have potential B and T cell epitopes that have been identified and generated using immunoinformatic techniques. Six B-cell epitopes in all were selected, including two for Rv0899 Mtb and four for the SARS-CoV-2 spike glycoprotein. Only nine epitopes were finally picked out of the 273 CTL ligands for Mtb's OmpA and the 37 predicted CTL ligands for the spike glycoprotein of SARS-CoV-2 that were found by the NetCTL 1.2 server. Although picking the epitopes requires time, the vaccine peptide may prevent SARS-CoV-2 and Mtb coinfection and may also strengthen the host's immune system^24^.

Artificial intelligence-based approaches including the support vector machine, hidden Markov model, and genetic algorithms have been used to address the aforementioned difficulties concerning B-cell and T-cell epitopes vaccine design^42^. Machine learning algorithms are perfect for data-driven sciences like genomics because their architecture automatically recognizes patterns in data Fields^43,44^. A protein's primary structure, which is a linear sequence of amino acids, contains the structural and functional data that the protein needs to function properly. Deep learning models like CNN and recurrent neural networks (RNN) have been successfully applied to protein sequences for several tasks, such as structure prediction or function. Although DL frameworks have been widely used in healthcare imaging^45–48^, there aren't many deep learning techniques available for forecasting B-cell and T-cell epitopes, and while some models appear effective at doing so based on their performances, they still struggle with accuracy. Since they are not capable of combining B-cell and T-cell predictions at a time, it is necessary to develop better models that will ease the tedious process of selecting B-cell and T-cell epitopes for building the vaccine. The contributions of this proposed method are:

- This study proposes a deep learning framework for combined predictions of B cell and T cell epitopes for the development of a TB multi-epitope type of vaccine. Using a DL architecture, it was possible to successfully replace the numerous essential predictions and thorough analyses of epitopes. Once a peptide sequence is fed into the DNN, it determines if it has the potential to become a subunit of the vaccine.
- Different machine learning models such as Artificial Neural Networks (ANN), Random Forests (RF), Naive Bayes, and Decision Trees were compared with the proposed method based on Deconvolutional Neural Networks (DCNN) coupled with Bidirectional Long Short-Term Memory (DCNN-BiLSTM).
- The DCNN-BiLSTM framework enables the initial step of reducing the number of prospective vaccine subunits, followed by additional assessment and vaccine design with the subunits expected to be B-cell and T-cell using trusted and well-liked in silico techniques.
- According to our research, the selected subunits from the six Mtb H37Rv antigens Rv1198, Rv2519, Rv3621c, Rv3344c, Rv0050, Rv3810 might be utilized successfully as possible vaccine candidates and would be employed in further experimental studies to eliminate TB.
- The H37Rv protein subunits with B-cell, HTL, and CTL epitopes that have been computationally verified were chosen to create the final MtbMEV construct. The top projected epitopes without having toxic or allergenic reactions but of course with promising antigenic properties were utilized.
- Numerous investigations were conducted to verify the applicability of the suggested vaccine, ranging from physiochemical aspects, secondary as well as tertiary structure analysis, interaction studies, and immune response modelling.

## Methodology

### Overview

**Figure 1:**
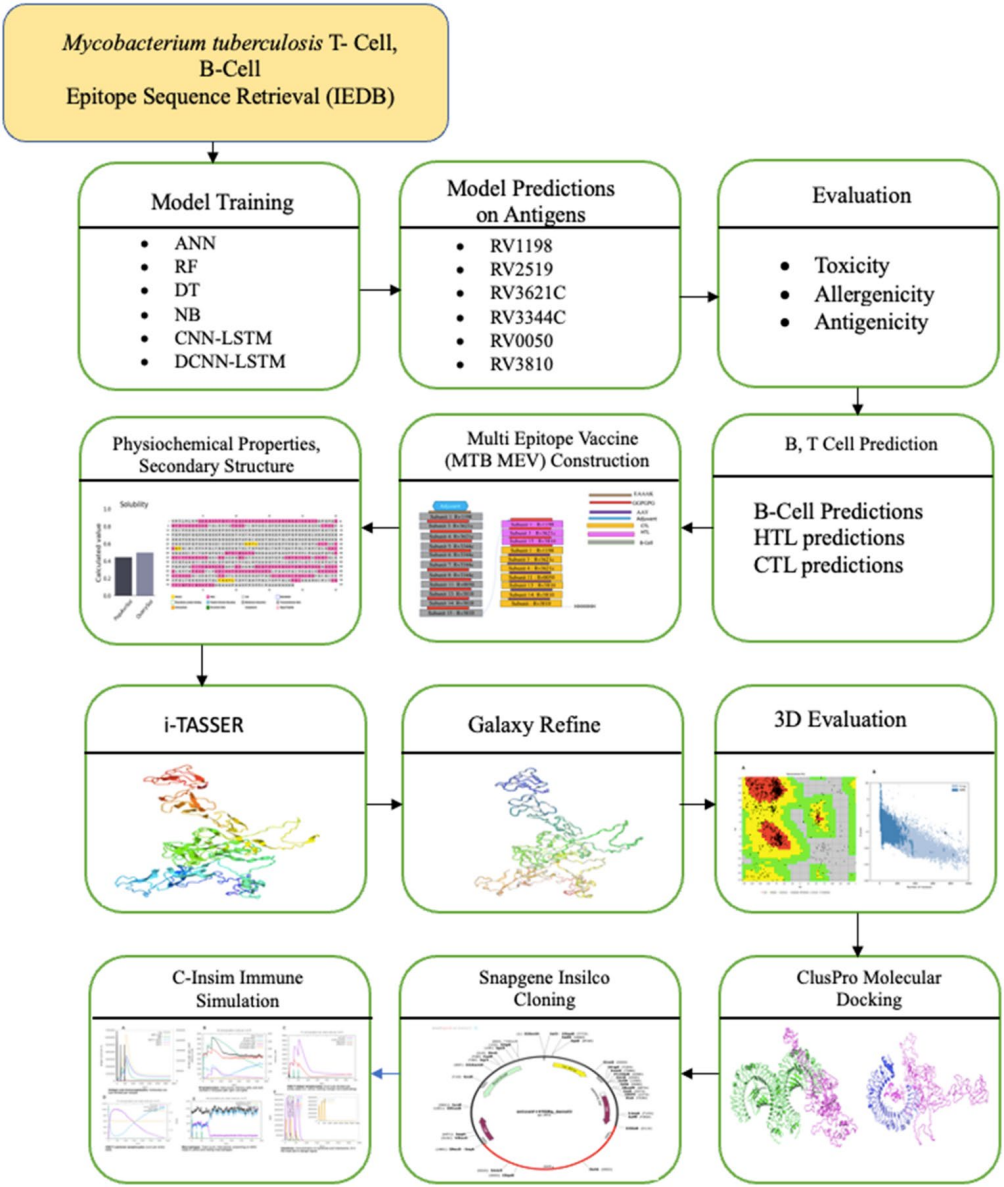
Overview of the proposed method.

In this study, the flow chart shown in Fig. 1 summarizes the whole work. In the beginning, Mtb epitopes were collected from the IEDB database for both positive and negative B-cells and T-cells containing MHC types 1 and 2. These datasets contain a few hundred epitopes so their cartesian products were formed T Χ B and B Χ T for both positive and negative TB to generate about 10 million epitopes. Different machine learning models including Bidirectional Long Short-Term Memory (Bi-LSTM), Deconvolutional Neural Networks (DCNN), Artificial Neural Networks (ANN), Random Forests (RF), Naive Bayes, and Decision Trees were trained on these datasets to develop models capable of predicting vaccine subunits for design of TB vaccine. The best-trained model DCNN-LSTM was used to predict probable vaccine subunits from six different Mtb antigens Rv1198, Rv2519, Rv3621c, Rv3344c, Rv0050, Rv3810 retrieved from mycobrowser database. These subunits were subjected to evaluations to predict toxicity, antigenicity, and allergenicity. Next, B-cell CTL and HTL TB epitopes were predicted from the subunits using different tools. The best subunits after the analysis were used to construct the final Multiepitope vaccine referred to as MtbMEV.

Following MtbMEV vaccine construction, secondary structure and various physiological properties such as aliphatic index, pI, GRAVY, half-life, and solubility were predicted. The 3D structure of the constructed vaccine was predicted and refined followed by 3D structure validation using i-TASSER, Galaxy Refine, and Ramachandran plot analysis respectively. To study the interaction of the constructed MtbMEV molecular docking was performed using two receptors TLR3 and TLR4. High interactions show the possibility of producing an immune response, this was confirmed by the level of innate and adaptive responses generated after immune response simulation. Finally, in-silico cloning confirmed the expression of the MtbMEV in *E. coli.* The results presented in this work can be utilized by scientists to study novel vaccines against TB successfully.

### Datasets

Epitopes unique to H37Rv *M. tuberculosis* were collected using the Immune Epitope Database and Analysis Resource (IEDB)^49^. B-cells and T-cells positive and negative epitopes were retrieved respectively (http://www.iedb.org/). Next, the mycobrowser database (https://mycobrowser.epfl.ch/) was used to collect six different *M. tuberculosis* H37Rv protein sequences Field^50^. These include Rv1198 (Accession No: P9WNJ5), Rv2519 (Accession No: Q79FD3), Rv3621c (Accession No: P9WHX3), Rv3344c (Accession No: L0TFC2), Rv0050 (Accession No: P71707), Rv3810 (Accession No: P9WIQ7). These proteins have undergone extensive research as the top candidates for the *M. tuberculosis* vaccine and exhibit the highest levels of antigenic, adhesin likelihood, and immune response activation. The knowledge provided by the research on these proteins suggests that they could be safe as vaccine candidates^51^.

### Models

#### Bidirectional long short-term memory (Bi-LSTM)

Bidirectional Long Short-Term Memory (Bi-LSTM) recurrent neural networks are a subclass of recurrent neural networks that can process sequential data in both forward and backward directions^52^. It is commonly used in research on speech recognition and natural language processing, where it is important to understand the context of each word. Two LSTM layers make up the Bi-LSTM model; one LSTM layer processes the input sequence forward, while the second LSTM layer processes it backwards. The outcome of each layer is combined to form the final output.

The forward and reverse states are calculated by the Bi-LSTM model using the following equations:

a. Forward LSTM equations:

1 $$ I_{t} = \, sigma\left( {W_{xi} x_{t} + \, W_{hi} h_{t - 1} + \, b_{i} } \right) $$

2 $$ f_{t} = \, sigma\left( {W_{xf} x_{t} + \, W_{hf} h_{t - 1} + \, b_{f} } \right) $$

3 $$ c_{t} = \, f_{t} * \, c_{t - 1} + \, i_{t} * \, tanh\left( {W_{xc} x_{t} + \, W_{hc} h_{t - 1} + \, b_{c} } \right). $$

4 $$ o_{t} = \, sigma\left( {W_{xo} x_{t} + \, W_{ho} h_{t - 1} + \, b_{o} } \right) $$

5 $$ h_{t} = \, o_{t} * \, tanh\left( {c_{t} } \right). $$

where *i*_*t*_*, f*_*t*_*,* and o t are the input, forget, and output gates, and *W* and *b* are the weights and biases of the LSTM layer. *x*_*t*_ is the input sequence at time *t. h*_*t*_ and *c*_*t*_ are the hidden state and cell state at time *t.*

b. Backward LSTM equations:

6 $$ i_{t}{\prime} = \, sigma(W_{xi}{\prime} x{\prime} t + W_{hi}{\prime} h_{t + 1}{\prime} + \, b_{i}{\prime} ). $$

7 $$ f_{t}{\prime} = \, sigma(W_{xf}{\prime} x_{t}{\prime} + \, W_{hf}{\prime} h_{t + 1}{\prime} + \, b_{f}{\prime} ) $$

8 $$ {\text{c}}_{t}{\prime} = \, f_{t}{\prime} * \, c_{t + 1}{\prime} + \, i_{t}{\prime} * \, tanh(W_{xc}{\prime} x_{t}{\prime} + W_{hc}{\prime} h_{t + 1}{\prime} + b_{c}{\prime} ) $$

9 $$ o_{t}{\prime} = \, sigma(W_{xo}{\prime} x_{t}{\prime} + \, W_{ho}{\prime} h_{t + 1}{\prime} + \, b_{o}{\prime} ) $$

10 $$ h_{t}{\prime} = \, o_{t}{\prime} *tanh\left( {c_{t}{\prime} } \right) $$

where *x'*_*t*_ is the input sequence at time *t* in the backward direction, *h'*_*t*_ and *c'*_*t*_ are the hidden state and cell state at time *t* in the backward direction, *i'*_*t*_*, f'*_*t*_, and *o'*_*t*_ are the input, forget, and output gates, and *W'* and *b'* are the weights and biases of the backward LSTM layer.

#### Convolutional neural networks (CNN)

The ability of Convolutional Neural Networks (CNN) to automatically learn and extract complex hierarchical features from grid-like input, particularly images, and sequences, has revolutionized the area of image analysis. A CNN's design consists of several layers, each of which helps the network recognize intricate patterns in its input data^53^.

Convolutional layers, which are at the heart of CNN, are where learnable filters, often referred to as kernels, are convolved with the input data to produce feature maps that capture local patterns. By gradually extracting characteristics of increasing complexity, the network can recognize edges, textures, and more abstract ideas. An activation function, frequently the Rectified Linear Unit (ReLU), follows each convolutional process, introducing non-linearity and enhancing the representation.

The convolution operation can be represented as follows:

$$Y[{\text{i}},j]={\sum }_{k,l}\left(X\left[i+k,j+l\right]\left({W}_{\left[k,l\right]}\right)\right)+bY\left[i,j\right]$$

where.

- *X* is the input data.
- *Y* is the output feature map.
- *W* is the learnable filter (kernel).
- *b* is the bias term.

Pooling layers are used to minimize computational complexity and spatial dimensions. These layers downsample the feature maps by combining data from nearby areas. Due to the translation invariance introduced by this downsampling, the network can detect patterns independent of their exact spatial location. Fully connected layers are then used to combine the hierarchical information collected from convolutional and pooling layers, producing final predictions based on high-level representations.

#### Deconvolution neural network

CNN transposition^54^, also known as deconvolution or transposed convolution, is essential for tasks like creating an image and comprehending how CNN operates. Transposed convolutions, in contrast to conventional convolutions, essentially "upsample" data by mapping smaller inputs to larger outputs.

Deconvolutional Neural Networks (DCNN) offer a unique method for improving classification accuracy in the area of predicting epitopes within TB sequences. DCNNs, which were first created for image generation and super-resolution, may be skillfully repurposed to effectively handle the particular difficulties associated with epitope categorization in data.

#### Artificial neural networks (ANN)

The topology of the Artificial Neural Networks (ANN)^55^, a fundamental machine learning tool, was modelled after the linked neurons of the human brain. ANNs excel in classifying epitopes within TB sequences by recognizing complex patterns. They are made up of input, hidden, and output layers, which are layers of interconnected nodes. The network may learn complicated associations because of the activation functions that neurons apply.

#### Random forest

Random Forests (RF)^56^, an ensemble learning method, is a potent tool. RF reduces overfitting and improves generalization by building numerous decision trees using a random sample of characteristics and data points. Voting or averages are used to combine decision trees' collective wisdom during prediction. This method, which is renowned for its adaptability and interpretability, provides insights into the significance of the features and performs well with noisy or complicated data.

#### Naive Bayes

The probabilistic classification technique Naive Bayes (NB)^57^ develops predictions based on observable characteristics, it uses conditional probability and Bayes' theorem. Calculations are made easier and training is expedited by the naive assumption of feature independence. Probability updates are guided by Bayes' theorem, which enables the algorithm to identify the most likely class for a given data. Naive Bayes is effective for the analysis of immunological data despite its simplicity because it performs well in high-dimensional feature spaces. The influence of the independence assumption can be reduced by using expert insights and domain knowledge to increase its efficiency.

#### Decision trees

Decision Trees (DT)^58^ simplify difficult selections into smaller, more manageable possibilities and arrange them in a tree-like framework. Decision Trees produce precise class assignments by choosing characteristics that reduce entropy through splits. The interpretability of the model is improved by its visual representation and feature significance insights. While ensembles like Random Forests and Boosting improve prediction accuracy, pruning strategies reduce overfitting.

### Prediction of B-cell epitopes

The synthesis of vaccines depends heavily on B-cell epitopes since they are essential for inducing a humoral immune response, which in turn drives B cells to generate antibodies. Four webservers, BepiPred-2.0^17^, BcePred^59^, ABCpred^60^, and SVMTrip^61^, were used to predict linear B-cell epitopes on vaccine components. BepiPred-2.0, which is available at (https://services.healthtech.dtu.dk/services/BepiPred-2.0/), predicts B-cell epitopes utilizing random forest algorithms using information gathered from 3D structures that have been solved and a sizable collection of linear epitopes obtained from the IEDB. The ABCpred service, which is available at (http://crdd.osdd.net/raghava/abcpred/ ), predicts B cell epitope(s) in an antigen sequence using a recurrent neural network with pre-set length patterns of 20 residues. A novel method called SVMTrip, available at (http://sysbio.unl.edu/SVMTriP/index.php), leverages sequence input from the IEDB database to predict antigenic epitopes. To enhance prediction performance, it applies a Support Vector Machine (SVM) on Tri-peptide similarity and Propensity scores (SVMTriP). Additionally, depending on the physicochemical properties of proteins, BCPreds, which can be found at (http://crdd.osdd.net/raghava/bcepred/), are employed. A kernel-based technique for prediction is also the foundation of the SVM model employed by BCPreds.

### Predicting CTL epitopes

The default settings for the NetCTL1.2 server (https://services.healthtech.dtu.dk/services/NetMHCpan-4.1/), such as the TAP transport efficiency, weight on C terminal cleavage, and threshold for epitope identification were utilized for forecasting CTL epitopes. The site predicts how peptides with a given sequence would bind to any MHC molecule using ANNs. The system is trained using over 850,000 quantifiable peptides from Mass-Spectrometry Eluted Ligands and Binding Affinity measurements^18^. Nine-residue CTL epitopes were examined; these epitopes are identified by HLA class-I supertypes such as B7, B8, B27, B39, B44, B58, B62, A1, A2, A3, A24, and A26. To be used in future vaccines, only those epitopes that were deemed to be strong binders were chosen.

### Predicting HTL epitopes

The HTL epitopes that HLA Class II DRB1 alleles recognize are 15 residues long and can be predicted using the NetMHCIIpan-4.0 server, which is accessible at (https://services.healthtech.dtu.dk/services/NetMHCIIpan-4.0/. Predictions were restricted by a pre-set threshold. The service predicts the likelihood of a specific peptide's binding to each MHC II molecule using ANNs. The enormous dataset of over 500,000 observations of binding affinity and eluted ligand mass spectrometry covers the three human MHC class II isotypes HLA-DP, HLA-DQ, and HLA-DR together with the mouse molecules (H-2)^62^.

### Epitope’s toxicity, allergenicity, and antigenicity prediction

Before the development of the vaccine candidate, all selected subunit candidates had been assessed for their anticipated allergenicity, toxicity, and antigenicity. To predict the toxicity, the ToxinPred server (https://webs.iiitd.edu.in/raghava/toxinpred/index.html) was employed. With the help of this program, users may forecast the toxicity of their peptides. It creates all feasible mutants of the specified sequences and evaluates their toxicity coupled with other physicochemical characteristics such as pI, charges, and hydropathicity^63^. Estimating allergenicity was done using the AllerTop V. 2.0 web server. The AllerTop which can be found at (https://www.ddg-pharmfac.net/AllerTOP/index.html) uses an auto cross-covariance (ACC) approach to assess how allergenic a protein is. It takes into account factors such as helix-forming propensity, strand-forming propensity, hydrophobicity, molecular size, and the relative abundance of amino acids. The application classifies the proteins based on a training set that consists of 2427 identified allergens from diverse species and 2427 non-allergens using the k-nearest neighbour technique (kNN)^64^. VaxiJen found at (http://www.ddg-pharmfac.net/vaxijen/VaxiJen/VaxiJen.html) can predict a protein's antigenicity in a way that is independent of alignment using the protein's physicochemical characteristics. A set of data for bacteria, viruses, and tumours were used to train the program. For each collection, 100 known antigens and 100 non-antigens were included to overcome the limitations of alignment-based approaches^65^.

### Design of the MtbMEV vaccine

A MtbMEV consisting of 21 different epitopes was designed. These epitopes are from the six proteins of *Mtb* H37Rv. Griselimycin obtained from PDB was used as an adjuvant boot immune response^66^. It was attached to the amino (N) terminus of the multi-subunit sequence by an EAAAK linker^67^. The GPGPG linkers bind eleven eleven B-cell epitope subunits and three HTL epitopes. Seven CTL epitopes were connected by AAY linkers and finally, a 6xHis tag was inserted at the C-terminal^68^. In other to reduce the probability of autoimmunity, protein similarity to human proteins was evaluated using BLAST. The UniProtKB Human database received the vaccine sequence to carry out the blast research (https://www.ebi.ac.uk/Tools/sss/ncbiblast/).

### Predictions of solubility, physiochemical characteristics, and secondary structure

It was possible to forecast the physicochemical properties of the vaccine constructs, including their in vitro and in vivo half-lives, amino acid composition, instability, aliphatic index, theoretical isoelectric point (pI), using Expasy Protparam found at https://web.expasy.org/protparam/^69^. A protein's anticipated instability index determines whether it is stable or unstable; if it is less than 40, it is considered stable, whereas proteins with a value higher than 40 fall into the group of unstable proteins. The aliphatic index of a protein measures how much space isoleucine, leucine, valine, and alanine the aliphatic side chains occupy. By dividing the total hydropathy for all of the amino acid residues in the protein by the total number of residues, the grand average of hydropathy was calculated^30^. Additionally, the Protein-Sol server located at (https://protein-sol.manchester.ac.uk) employed a population average (PopAvrSol) of 0.45 to assess the solubility of a multi-epitope vaccine, with values greater than 0.45 suggesting improved solubility while a lower value will be less soluble^70^. The secondary structure of our final vaccine is predicted using PSIPRED, which may be accessed at (http://bioinf.cs.ucl.ac.uk/psipred/)^71^. We also make use of the RaptorX Property web server, which is accessible at (http://raptorx.uchicago.edu/StructurePropertyPred/predict/), to anticipate the solvent accessibility (ACC)^72^.

### Vaccine’s 3D structure prediction

The vaccine's tertiary or three-dimensional (3D) model was made using the homology modelling software I-TASSER (Iterative Threading Assembly Refinement) platform (https://seq2fun.dcmb.med.umich.edu//I-TASSER/). It is a unified platform that uses the Protein Data Bank (PDB) to discover similar structural patterns to computationally predict protein structure and function based on sequence, structure, and function. I-TASSER initially produces 3D atomic models from an amino acid sequence by employing a variety of threading alignments and iterative structure assembly simulations. An accurate topology is demonstrated by a template modelling TM score > 0.5, while a random similarity is shown by a TM score < 0.17^73^.

### Refinement of 3D structure

Using the GalaxyRefine web server (http://galaxy.seoklab.org/cgi-bin/submit.cgi?type=REFINE) the vaccine peptide's 3D model will be enhanced. Based on refining techniques that were successfully tested in CASP10-based refinement studies, the GalaxyRefine server was created and accomplished the structure's relaxation by repacking and molecular dynamics modelling. When applied to modern protein structure prediction models this method can improve the overall standard of local as well as global structures. The Molprobity score, GDT-HA score, RMSD score, and Clash score are used to assess the quality of the revised model^74^.

### Validation of 3D structure of vaccine

To produce vaccines, it is crucial to validate the tertiary structure since it might highlight problems with the predicted model. We evaluated and got a Ramachandran plot which displays the number of residues in either allowed or prohibited domains^75^. Ramachandran plot for the 3D validation was conducted using VADAR^76^ (http://vadar.wishartlab.com/index.html?), and PROCHECK^77^ servers https://www.ebi.ac.uk/thornton-srv/databases/pdbsum/Generate.html. The quality and possibility of the 3D model's inaccuracy were checked using the ProSA-web server (https://prosa.services.came.sbg.ac.at/prosa.php). If Z scores are beyond the range of natural protein, the structural design is likely to include faults^78^. Furthermore, ERRAT^79^ and VERIFY 3D^80^ were further utilized to validate the 3D structure of the multiepitope vaccine.

### Molecular docking

Utilizing the Cluspro docking service (https://cluspro.bu.edu), molecular docking analysis was used to analyze the vaccine's interaction pattern with TLR3 alongside TLR4. The 3D structures of human TLR3 along with TLR4 receptors with IDs: 3fxi and 2z63, respectively, were obtained using Protein Data Bank (PDB). The service provides cluster ratings based on rigid docking and pairwise RMSD energy reduction, selecting the best-docked model with the lowest energy weight score after sampling billions of conformations^81^. Using the PyMOL visualization technology, the best vaccine plus TLR3 complex and vaccine plus TLR4 complex models were selected and visualized.

### In silico cloning and codon optimization

The Java Codon Adaptation Tool (JCat) service available at (http://www.jcat.de) is used for codon optimization^82^. Codon optimization is essential since the genetic code is degenerate and the majority of amino acids may be translated by many codons. Although more than 0.8 can be considered a favourable result since it exposes codon usage biases, the ideal CAI score is 1.0^83^. The sequence's GC content must be between 30 and 70%; any value outside of this range has a detrimental effect on the efficiency of transcription and translation. The improved codon sequence is added to the pREP4 vector to calculate the levels of protein expression in *E. coli.* using the SnapGene 5.1.5 program available at (https://www.snapgene.com). Finally, the vaccine was inserted at restriction sites ApoI (1981) and BstEII (4115) into the pREP4 vector.

### Immune response simulation via C-IMMSIM server

The C-ImmSim internet simulation service^84^ was used to provide information about the profile of the immunological response following vaccination. C-ImmSim will assess a mammalian immune system’s humoral and cellular reaction to the vaccine formulation (https://kraken.iac.rm.cnr.it/C-IMMSIM/index.php?page=1). In this study, reactivity was evaluated after three injections of 1000 antigens, spaced by four weeks. 1, 75, and 150 were the utilized periods. There were no changes made to any simulation settings.

## Results

Predicting Mtb epitopes is a key step in creating efficient vaccines and diagnostic tools, and this was the focus of this study. To do this, we compared the performance of six machine learning models over two datasets, each having 2 million and 8 million sequences, respectively. To evaluate the models' ability to correctly classify epitopes, important performance parameters including accuracy, sensitivity, specificity, F1-score, and AUC were used.

The DCNN-LSTM model performed exceptionally well on the 2 million sequence dataset, achieving accuracy rates of 98.86%, sensitivity rates of 98.90%, specificity rates of 98.82%, F1-score rates of 98.86%, and an AUC value of 0.9996 see Table 1. The impressive findings of this model demonstrate its reliability in classifying both positive and negative epitopes. The Random Forest (RF) and Decision Tree (DT) models also performed well, achieving accuracy rates of 97.25%, sensitivity rates of 99.06%, specificity rates of 90.02%, F1-score rates of 98.29%, and AUC rates of 0.9863 and 94.90%, sensitivity rates of 96.69%, specificity rates of 87.76%, respectively.

**Table 1:** Model performance trained with 2 million epitope sequences.

Models	Accuracy	sensitivity	Specificity	F1-Score	AUC
DCNN-LSTM	0.9886	0.989	0.9882	0.9886	0.9996
CNN-LSTM	0.8224	0.9429	0.701	0.7979	0.9354
RF	0.9725	0.9906	0.9002	0.9829	0.9863
DT	0.949	0.9669	0.8776	0.9681	0.9225
NB	0.602	0.6674	0.6043	0.5928	0.7083
ANN	0.8434	0.9696	0.3405	0.9083	0.8622

However, when compared to other models, the CNN-LSTM model showed lesser specificity (70.10%) and accuracy (82.24%). Nevertheless, it made up for this with better sensitivity (94.29%), exhibiting its ability to classify real positive epitopes. The model has an F1-score of 79.79% and an AUC of 0.9354. However, the Naive Bayes (NB) and Artificial Neural Network (ANN) models performed poorly on this dataset, with NB achieving accuracy of 60.20%, sensitivity of 66.74%, specificity of 60.43%, F1-score of 59.28%, and AUC of 0.7083, and ANN achieving accuracy of 84.34%, sensitivity of 96.96%, specificity of 34.05%, F1-score of 90.

The DCNN-LSTM model maintained its superior efficiency concerning the larger 8 million sequence dataset Table 2, achieving an accuracy of 99.46%, sensitivity of 97.60%, specificity of 99.92%, F1-score of 99.66%, and an AUC of 0.9996. Its higher performance on the large dataset demonstrates that it is capable of making accurate predictions of TB epitopes. With an accuracy of 96.87%, sensitivity of 96.17%, specificity of 97.57%, F1-score of 96.85%, and an AUC of 0.9932, the RF model performed well. The DT model performed similarly, displaying stable performance with an accuracy of 94.96%, sensitivity of 94.67%, specificity of 95.25%, F1-score of 94.95%, and AUC of 0.9552.

**Table 2:** Model performance trained with 8 million epitope Sequences.

Models	Accuracy	sensitivity	Specificity	F1-Score	AUC
DCNN-LSTM	0.9946	0.976	0.9992	0.9966	0.9997
RF	0.9687	0.9617	0.9757	0.9685	0.9932
DT	0.9496	0.9467	0.9525	0.9495	0.9552
ANN	0.8235	0.8164	0.8306	0.8223	0.902
NB	0.6082	0.5443	0.6721	0.5816	0.667
CNN-LSTM	0.9144	0.9753	0.6716	0.948	0.939

On the larger dataset, the ANN model particularly showed progress, achieving an accuracy of 82.35%, a sensitivity of 81.64%, a specificity of 83.06%, an F1-score of 82.23%, and an AUC of 0.9020. An accuracy of 91.44%, sensitivity of 97.53%, specificity of 67.16%, F1-score of 94.80%, and AUC of 0.9390 were attained by the CNN-LSTM model on the bigger dataset.

With an accuracy of 60.82%, sensitivity of 54.43%, specificity of 67.21%, F1-score of 58.16%, and AUC of 0.6670, the Naive Bayes (NB) model failed to provide results that were able to compete with the larger dataset.

**Figure 2:**
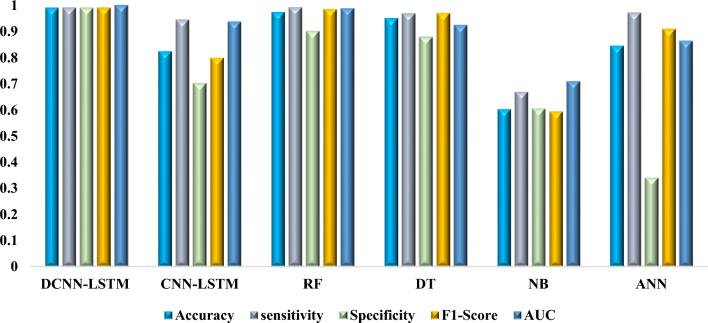
The figure presents the performance of six machine learning models on a dataset containing 2 million epitope sequences, focusing on accuracy, sensitivity, specificity, F1-score, and AUC.

**Figure 3:**
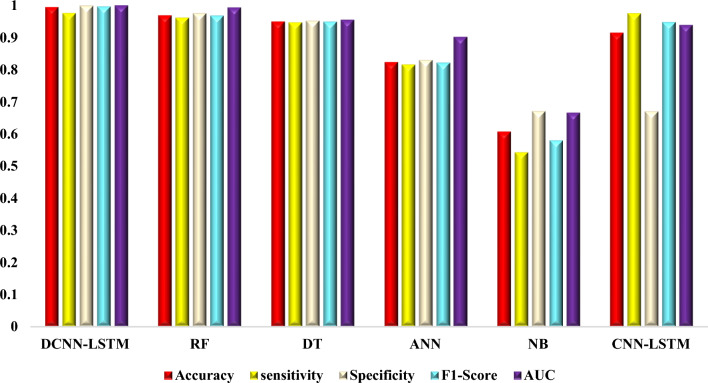
The performance of six machine learning models on a dataset containing 8 million epitope sequences was evaluated, focusing on accuracy, sensitivity, specificity, F1-score, and AUC.

On both datasets, the DCNN-LSTM model consistently outperformed other models, demonstrating its reliability and efficiency in identifying TB epitopes. The RF and DT variants performed effectively as well. It was clear that dataset size had an impact on model performance, with certain models significantly outperforming smaller datasets as shown in Figs. 2 and 3. The selection of appropriate machine learning models for tasks requiring epitope prediction is made easier thanks to the insights provided by these findings for academics and professionals working in the field of developing TB vaccines and diagnostics. Future work might concentrate on improving model performance and investigating the use of deep learning approaches for epitope prediction in infectious diseases like TB.

### B-cell predictions

For the analysis of epitopes that are B-cell type, all the 29 subunits that were predicted as epitopes by our proposed model were utilized. Among the 29 subunits, 15 subunits were predicted as B-cell epitopes using different tools. As shown in Table 3 these subunits were used by the Bepipred to predict B-cell epitopes. Other tools such as ABCpred, SVMStrip and BcePred were also utilized. Until two of these tools forecasted the subunits as B-cell containing, only then they can be categorized as a B-cell epitope. The peptide sequences as shown in Table 3 came from different proteins of the Mtb. Immunoglobulin or antibodies can attach to certain regions of an antigen called B-cell epitopes, which then cause the B-cells to produce an immunological response^85^. In total, all the six proteins used in this research contain B-cell epitopes showing the possibility of high immunological response.

**Table 3:** B-cell epitopes were predicted using peptide subunits identified by the proposed DCNN-LSTM model.

Protein	Subunits	Start	End	Peptide Predicted by model	Bepipred
Rv1198	1	60	89	FQVIYEQANAHGQKVQAAGNNMAQTDSAVG	YEQANAHGQKVQAAGNNMAQTDS
Rv2519	2	330	359	YAVSGPGNVVTTDLPGQLNEGTLIDIPGGY	PGNVVTTDLPGQLNEGTLI
Rv3621c	3	150	179	AMYGYAGASAAATQLSPFNPAAQTINPAGL	GASAAATQLSPFNPAAQTIN
Rv3621c	4	300	329	GAKAAGEAAKALPAAVPAIPSAGLSGVAGA	AKALPAAVPAIPSAGLSGVAG
Rv3344c	5	180	209	NGGAGGNATGSGGKGGAGGNGGDGSFGATS	GGNATGSGGKGGAGGNGGDGSFG
Rv3344c	6	330	359	GFGGDGGQGGPNGGGTVGTVAGGGGNGGVG	DGGQGGPNGGG
Rv3344c	7	390	419	NGGLGGAGGGGGNAPDGGFGGNGGKGGQGG	GAGGGGGNAPDGGFGGNGGKGG
Rv3344c	8	420	449	IGGGTQSATGLGGDGGDGGDGGNGGNSGAK	TQSATGLGGDGGDGGDGGNGGNS
Rv3344c	9	450	479	AGGAGGKGQAGQPNSGTEPGFGGDGGLGGA	GGKGQAGQPNSGTEPGFGGDGG
Rv0050	10	360	389	GLGYQVDSSPLTVDGIKITNVEGEGCGTCN	QVDSSPLTVDGIKITNVEGEGC
Rv0050	11	420	449	AHQAGIASSFPGVAHTLSEDGKGGPPNNGI	ASSFPGVAHTLSEDGKGGPPN
Rv0050	12	600	629	DGALKGTSNETFPKPTEVGGYAGVPPPPPP	KGTSNETFPKPTEVGGY
Rv3810	13	60	89	ALSQGLSQFGINIPPVPSLTGSGDASTGLT	GLSQFGINIPPVPSLTGSGDAST
Rv3810	14	150	179	LTSPTGATPGLTSPTGLDPALGGANEIPIT	GATPGLTSPTGLDPALGGANE
Rv3810	15	240	269	VLMPSIMQAVQNGGAAAPAASPPVPPIPAA	QAVQNGGAAAPAASPPVPP

### CTL epitopes prediction

NetMHCpan-4.1 web server was used for the analysis of MHC-I or CTL epitopes. Different subtypes of MHC-I were selected and for each subunit, the epitopes were predicted to contain both strong and weak binders. As shown in the Table 4 each peptide sequences were able to bind to some particular group of subtypes and the total HLA score is presented. Among the 15 subunits that were predicted as B-cell epitopes, 13 were forecasted as MHC-I epitopes. Based on the HLA score, seven subunits with scores above 4.0 were selected as the possible subunits for the vaccine construction.

**Table 4:** Prediction of MHC-I or CTL Epitopes from peptide subunits identified by proposed DCNN-LSTM model alongside their HLA subtypes using NetMHCpan-4.1 server.

	Peptide sequence	No. of epitopes	HLA subtypes	HLA score
1	FQVIYEQANAHGQKVQAAGNNMAQTDSAVG	8	HLA-A*03:01, HLA-B*07:02, HLA-B*39:01, HLA-B*40:01, HLA-B*15:01	2.254
2	YAVSGPGNVVTTDLPGQLNEGTLIDIPGGY	15	HLA-A*01:01, HLA-A*03:01, HLA-A*26:01, HLA-B*07:02, HLA-B*40:01, HLA-B*15:01	4.762
3	AMYGYAGASAAATQLSPFNPAAQTINPAGL	15	HLA-A*02:01, HLA-A*24:02, HLA-A*26:01, HLA-B*07:02, HLA-B*39:01, HLA-B*40:01, HLA-B*58:01, HLA-B*15:01	4.307
4	GAKAAGEAAKALPAAVPAIPSAGLSGVAGA	17	HLA-A*02:01, HLA-B*07:02, HLA-B*08:01, HLA-B*39:01, HLA-B*40:01	4.58
5	GFGGDGGQGGPNGGGTVGTVAGGGGNGGVG	3	HLA-B*07:02	0.510
6	AGGAGGKGQAGQPNSGTEPGFGGDGGLGGA	2	HLA-B*15:01, HLA-B*40:01	0.592
7	GLGYQVDSSPLTVDGIKITNVEGEGCGTCN	10	HLA-A*01:01, HLA-A*02:01, HLA-A*24:02, HLA-B*07:02, HLA-B*39:01	2.149
8	IAEALKMSLNTSYYRLMLKLNGGPQAVADA	40	HLA-A*01:0, HLA-A*02:01, HLA-A*03:01, HLA-A*24:02, HLA-A*26:01, HLA-B*07:02, HLA-B*08:01, HLA-B*39:01, HLA-B*40:01, HLA-B*58:01, HLA-B*15:01	11.318
9	AHQAGIASSFPGVAHTLSEDGKGGPPNNGI	24	HLA-A*02:01, HLA-A*03:01, HLA-A*24:02, HLA-A*26:01, HLA-B*07:02, HLA-B*08:01, HLA-B*27:05, HLA-B*39:01, HLA-B*40:01, HLA-B*58:01, HLA-B*15:01	8.289
10	DGALKGTSNETFPKPTEVGGYAGVPPPPPP	18	HLA-A*01:01, HLA-A*02:01, HLA-A*03:01, HLA-A*26:01, HLA-B*07:02, HLA-B*08:01, HLA-B*39:01, HLA-B*40:01, Allele HLA-B*58:01, HLA-B*15:01	4.855
11	ALSQGLSQFGINIPPVPSLTGSGDASTGLT	12	HLA-A*02:01, HLA-A*24:02, HLA-A*26:01, HLA-B*08:01, HLA-B*39:01, HLA-B*40:01, HLA-B*58:01, HLA-B*15:01	2.793
12	LTSPTGATPGLTSPTGLDPALGGANEIPIT	10	HLA-A*02:01, HLA-B*07:02, HLA-B*39:01	3.472
13	VLMPSIMQAVQNGGAAAPAASPPVPPIPAA	17	HLA-A*02:01, HLA-B*07:02, HLA-B*58:01	5.609

### HTL epitope predictions

The NetMHCIIpan-4.0 server was used for predicting the MHC-II binding possibility of the epitopes. Among the 15 subunits analysed as B-cell epitopes, 7 subunits were MHC-II binders as shown in Table 5. The ones selected as components of the final vaccine are those with HLA scores above 3.5, therefore 3 subunits were included as HTL epitopes.

**Table 5:** Prediction of MHC-II or HTL-epitopes with NetMHCIIpan-4.0 server from peptide subunits identified by proposed DCNN-LSTM model alongside their subtypes.

	Fragments	HTL epitopes	Subtypes	HLA score
1	FQVIYEQANAHGQKVQAAGNNMAQTDSAVG	31	HLA-DRB1*04:01, HLA-DRB1*09:01, HLA-DRB1*10:01,HLA-DRB1*01:01,HLADRB1*07:01,HLA-DRB1*08:01,HLA-DRB1*09:01,HLA-DRB1*10:01,HLA-DRB1*11:01,HLA-DRB1*15:01,HLA-DRB1*11:01,HLA-DRB1*16:01	15.786
2	YAVSGPGNVVTTDLPGQLNEGTLIDIPGGY	4	HLA-DRB1_0301	1.468
3	AMYGYAGASAAATQLSPFNPAAQTINPAGL	8	HLA-DRB1*09:01, HLA-DRB1*10:01, HLA-DRB1*01:01	4.719
4	GLGYQVDSSPLTVDGIKITNVEGEGCGTCN	7	HLA-DRB1_0401, HLA-DRB1_0101, HLA-DRB1_0301, HLA-DRB1_1401	3.525
5	AHQAGIASSFPGVAHTLSEDGKGGPPNNGI	1	HLA-DRB1*03:01	0.251
6	DGALKGTSNETFPKPTEVGGYAGVPPPPPP	5	HLA-DRB1_0701,HLA-DRB1_1401, HLA-DRB1_1501	2.1
7	VLMPSIMQAVQNGGAAAPAASPPVPPIPAA	1	HLA-DRB1_0401	0.331

### Prediction of epitope's toxicity, allergenicity, and antigenicity

In the process of vaccine construction, it is necessary to analyse each of the vaccine subunits for toxicity to make sure none of the components is going to be toxic or harm humans. Also, we want to design a vaccine with high antigenicity so that it will raise a high immune response. In other to be utilized as a subunit for the final vaccine allergenicity needs to be eliminated. Table 6 shows that none of the vaccine subunits is going to be toxic as predicted by the ToxinPred tool, further allergenicity reactions were assessed by AllerTop to finally classify them as safe to use in the final vaccine. The MW, PI, charge, hydrophilicity and hydrophobicity were shown in Table 6, 11 out of the 15 subunits were predicted as non-allergen and were antigenic according to the VaxiJen tool see Table 7. Although 15 B-cell epitopes were predicted by the deep learning model and Bepipred, some of the epitopes were allergenic and were therefore removed from the final vaccine. These epitopes were from subunit 2, subunit 9, subunit 10 and subunit 12. All other 11 epitope subunits are non-allergenic and were used in the final vaccine.

**Table 6:** Toxicity analysis of vaccine subunits and their various immunogenic features.

	Fragments	Toxin pred score	Toxicity	Hydropathicity	Hydrophilicity	charge	PI	MW
1	FQVIYEQANAHGQKVQAAGNNMAQTDSAVG	− 1.53	Non-toxin	− 0.45	− 0.18	− 0.50	5.33	3148.87
2	YAVSGPGNVVTTDLPGQLNEGTLIDIPGGY	− 1.31	Non-toxin	0.04	− 0.33	− 3.00	3.50	3018.80
3	AMYGYAGASAAATQLSPFNPAAQTINPAGL	− 0.93	Non-toxin	0.25	− 0.59	0.00	5.87	2925.69
4	GAKAAGEAAKALPAAVPAIPSAGLSGVAGA	1.25	Non-toxin	0.75	− 0.16	1.00	8.94	2545.35
5	NGGAGGNATGSGGKGGAGGNGGDGSFGATS	− 0.95	Non-toxin	− 0.59	0.07	0.00	6.19	2354.76
6	GFGGDGGQGGPNGGGTVGTVAGGGGNGGVG	− 0.75	Non-toxin	− 0.23	− 0.16	− 1.00	3.80	2331.85
7	NGGLGGAGGGGGNAPDGGFGGNGGKGGQGG	− 0.70	Non-toxin	− 0.68	0.05	0.00	6.19	2315.81
8	IGGGTQSATGLGGDGGDGGDGGNGGNSGAK	− 0.61	Non-toxin	− 0.73	0.26	− 2.00	3.94	2448.89
9	AGGAGGKGQAGQPNSGTEPGFGGDGGLGGA	− 1.01	Non-toxin	− 0.60	0.11	− 1.00	4.38	2487.02
10	GLGYQVDSSPLTVDGIKITNVEGEGCGTCN	− 0.43	Non-toxin	− 0.11	− 0.03	− 3.00	3.92	3027.79
11	AHQAGIASSFPGVAHTLSEDGKGGPPNNGI	− 0.52	Non-toxin	− 0.37	− 0.08	0.00	6.02	2887.57
12	DGALKGTSNETFPKPTEVGGYAGVPPPPPP	− 0.63	Non-toxin	− 0.74	0.12	− 1.00	4.68	2978.74
13	ALSQGLSQFGINIPPVPSLTGSGDASTGLT	− 1.05	Non-toxin	0.26	− 0.40	− 1.00	3.80	2886.67
14	LTSPTGATPGLTSPTGLDPALGGANEIPIT	− 1.26	Non-toxin	0.11	− 0.26	− 2.00	3.67	2820.60
15	VLMPSIMQAVQNGGAAAPAASPPVPPIPAA	− 1.55	Non-toxin	0.65	− 0.51	0.00	5.88	2824.78

**Table 7:** The allergenicity, antigenicity predictions and half-life of subunits.

	Subunits	ALLERTOP	Vaxijen	VaxiJen score	Half-life in-vitro (h)	Half-life in-vivo
1	FQVIYEQANAHGQKVQAAGNNMAQTDSAVG	Non-allergen	Antigen	1.1312	1.1	2 min
2	AMYGYAGASAAATQLSPFNPAAQTINPAGL	Non-allergen	Antigen	0.5022	4.4	> 10 h
3	GAKAAGEAAKALPAAVPAIPSAGLSGVAGA	Non-allergen	Antigen	0.9628	30	> 10 h
4	NGGAGGNATGSGGKGGAGGNGGDGSFGATS	Non-allergen	Antigen	3.2141	1.4	> 10 h
5	GFGGDGGQGGPNGGGTVGTVAGGGGNGGVG	Non-allergen	Antigen	2.8340	30	> 10 h
6	NGGLGGAGGGGGNAPDGGFGGNGGKGGQGG	Non-allergen	Antigen	3.0626	1.4	> 10 h
7	IGGGTQSATGLGGDGGDGGDGGNGGNSGAK	Non-allergen	Antigen	2.9305	20	> 10 h
8	AHQAGIASSFPGVAHTLSEDGKGGPPNNGI	Non-allergen	Antigen	0.8502	4.4	> 10 h
9	ALSQGLSQFGINIPPVPSLTGSGDASTGLT	Non-allergen	Antigen	1.1165	4.4	> 10 h
10	LTSPTGATPGLTSPTGLDPALGGANEIPIT	Non-allergen	Antigen	0.8978	5.5	2 min
11	VLMPSIMQAVQNGGAAAPAASPPVPPIPAA	Non-allergen	Antigen	0.5175	100	> 10 h

### Construction of multi-epitope vaccine

**Figure 4:**
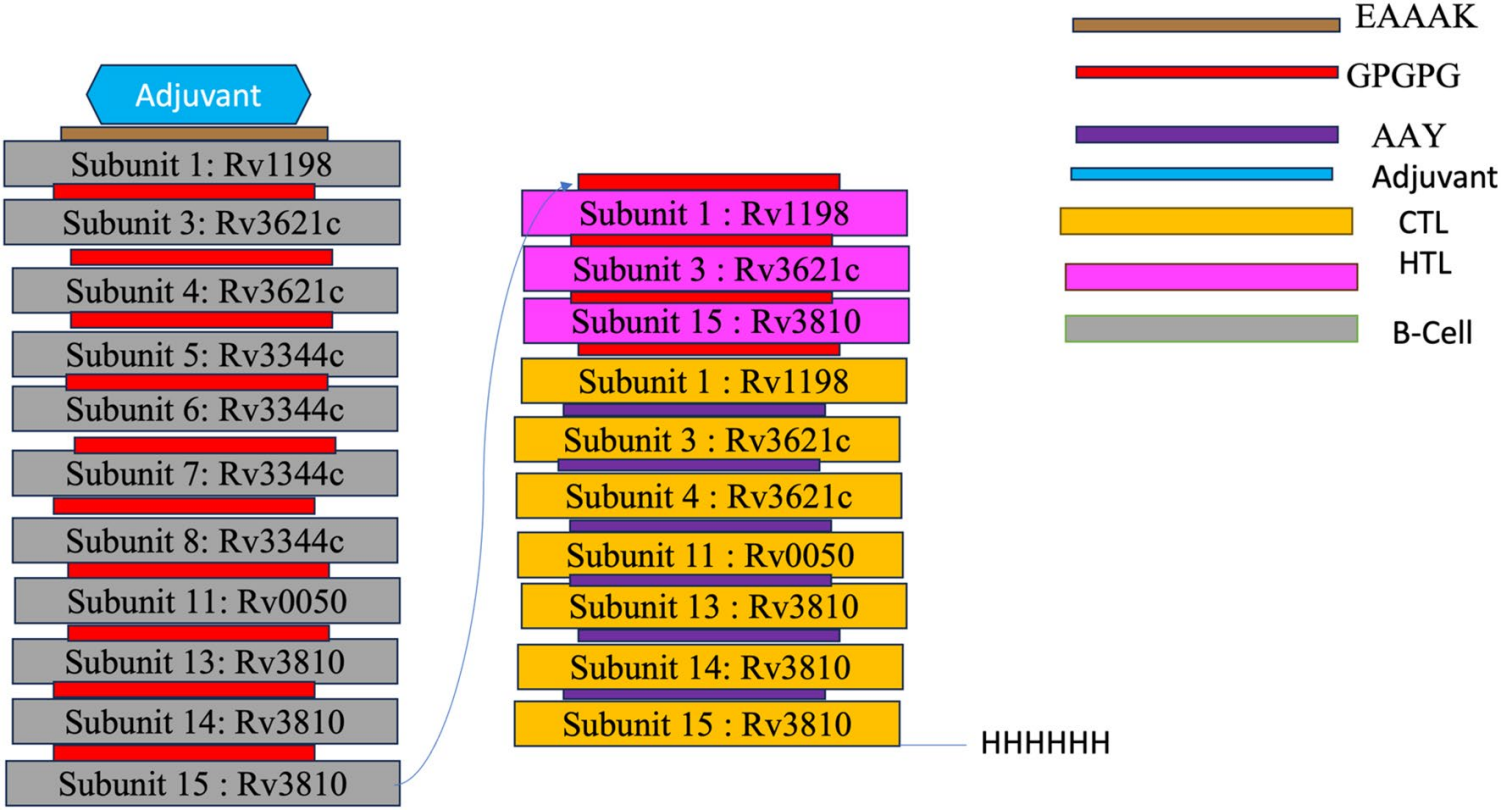
Multiepitope vaccine construction. A MtbMEV consisting of 21 different epitopes was designed. Griselimycin was attached to the amino (N) terminus by an EAAAK linker. The GPGPG linkers bind eleven B-cell epitope subunits and three HTL epitopes. Seven CTL epitopes were connected by AAY linkers and finally, a 6xHis tag was eventually inserted at the C-terminal.

A final MtbMEV with 738 amino acids was constructed in Fig. 4, consisting of 21 different epitopes. These epitopes are from the six proteins of *Mtb* H37Rv Rv1198, Rv2519, Rv3621c, Rv3344c, Rv0050, and Rv3810 retrieved from the mycobrowser database. This MtbMEV consists of an adjuvant Griselimycin (blue colour) obtained from PDB; 5ah2 to boot immune response. The adjuvant is linked to eleven B-cell epitopes (shown in grey) using the EAAAK linker. The B-cell epitopes are connected by a GPGPG linker (red colour). Next, three HTL epitopes (shown in magenta) are connected through a GPGPG linker to the CTL epitopes. Seven CTL epitopes (yellow colour) were also added to the MtbMEV through the GPGPG linker. The CTL epitopes were connected using the AAY linker (shown in purple). Finally, six histidine molecules were added at the end of the vaccine to serve as a tag for easy identification during purification. We use the Uniprot database to do a BLAST search on the vaccine components to rule out potential autoimmunity. None of the vaccine subunits that we finally chose to employ in the vaccine's development show much resemblance to the human proteome.

### Solubility, physiochemical properties, and secondary structure predictions

**Figure 5:**
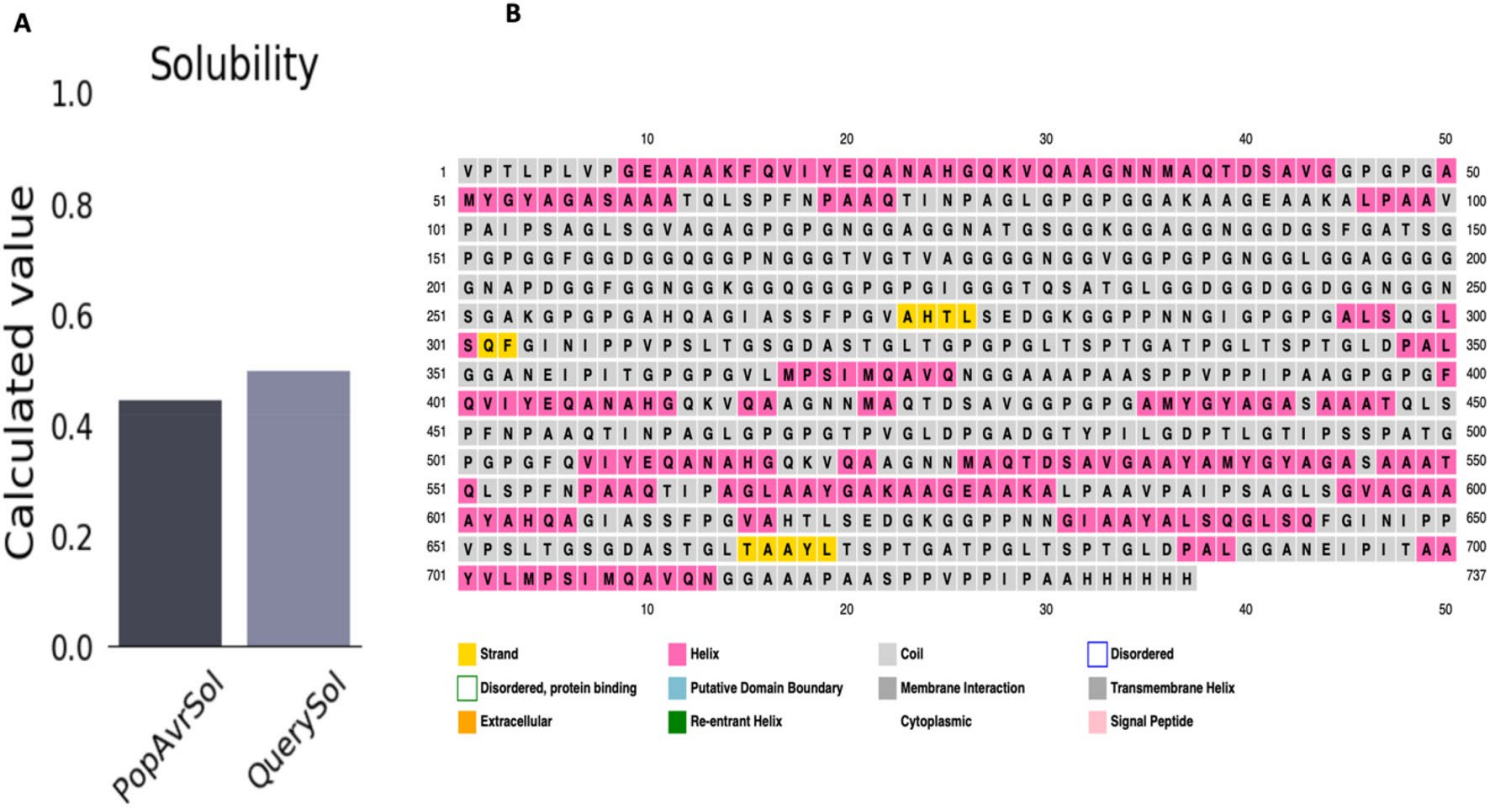
Prediction of vaccine constructs solubility and a secondary structure. (**A**) ProtSol’s evaluation of the designed MtbMEV solubility produced a result of 0.50. (**B**) Vaccine secondary structure prediction using the PSIPRED service.

The physicochemical properties of the vaccine were obtained from the Expasy tool. It has 738 amino acids, with 67,228.96 Da as molecular weight indicating that the vaccine will be antigenic. The half-life in-vitro in mammalian reticulocytes is > 100 h, also the in-vivo half-life in yeast is > 20 h and > 10 h in *Escherichia coli*. According to the instability index 33.47, the protein is classified as stable. The Aliphatic index is 62.70 while the pI is 5.08 and the GRAVY score − 0.077 was obtained. The protein is thermostable, as shown by the estimated aliphatic index of 62.70, the higher the value of the aliphatic index the more thermostable the protein. Also, the negative GRAVY score represents a protein's hydrophilic nature and its propensity to interact with water molecules. Finally, the Sol-Pro server was used to estimate the vaccine’s solubility, according to the predicted score of 0.500 (QuerySol) (Fig. 5A). It is said to be soluble with a score greater than 0.45 in the PopAvrSol; which is the population average for the experimental dataset. Secondary structure prediction of the vaccine was carried out by utilizing the PSIPRED server (Fig. 5B). It contains 87% coil, 10% beta, and 2% helix. This solvent analysis of the secondary structure by RaptorX server revealed good solvent accessibility with total amino acids divided into 63% that are been exposed, while 11% within medium range, and 25% are buried.

### 3-D structure of vaccine

**Figure 6:**
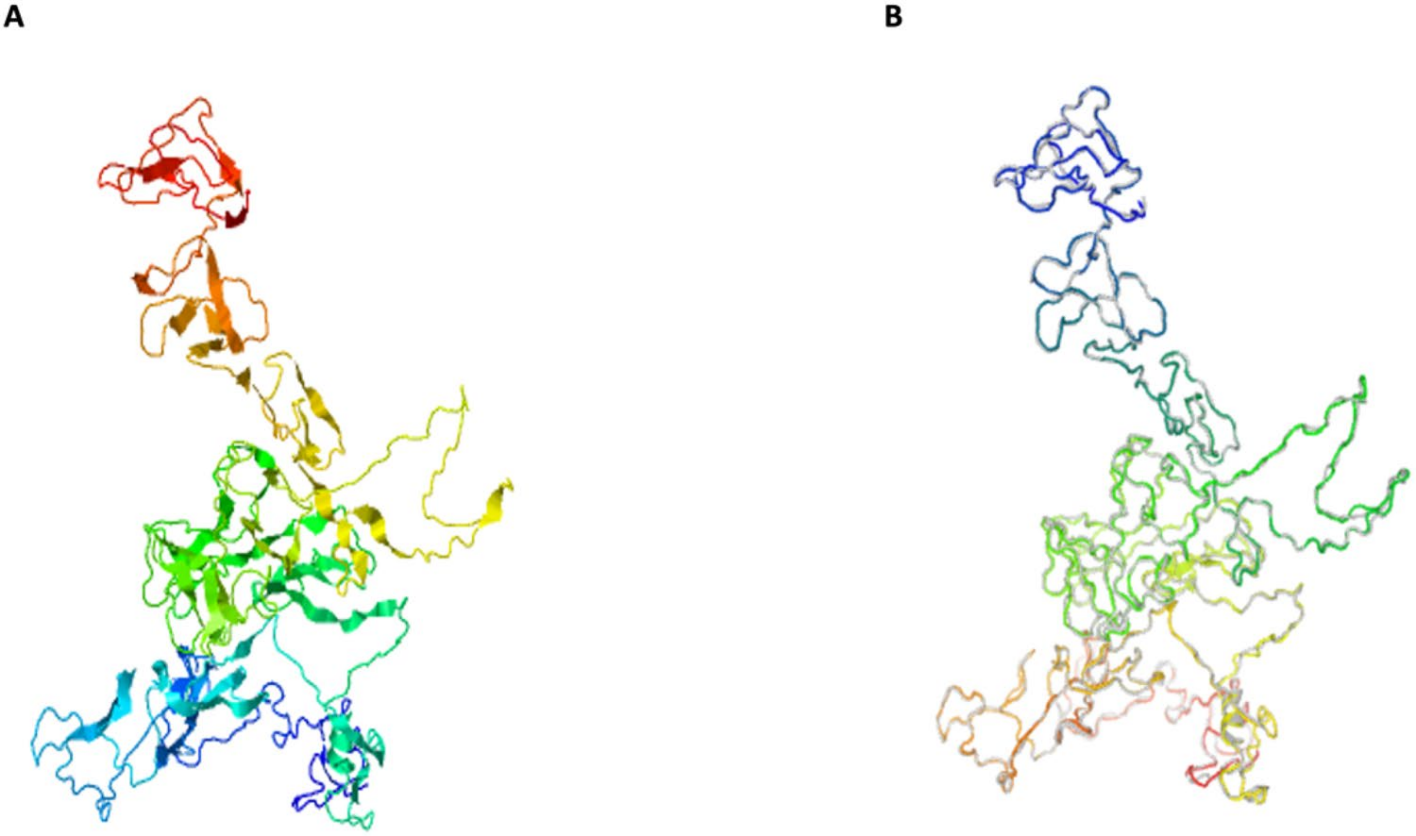
(**A**) MtbMEV anticipated 3D structure from the i-TASSER server and (**B**) the improved 3D model created by the Galaxy Refine server.

Utilizing the structural templates from the PDB database, the I-TASSER server does modelling. Even while the server has access to a vast array of possible template alignments, it only ever chooses the most correct ones. Here with confidence ratings (C-scores) ranging from (− 3.11 to − 1.47), the I-TASSER web server predicted five 3-D structures of the desired vaccine from 10 threading templates. The normal C score range is between − 5 and 2, with higher numbers signifying better accuracy. The model's top structure with a C value below − 1.47 was chosen for further examination. It was chosen as the best because Fig. 6A shows that it has the best characteristics for a multi-epitope tertiary structure. This structure has a projected TM-score of 0.53 ± 0.15 and a predicted root-mean-square deviation (RMSD) score of 11.8 ± 4.5 Å.

### Refinement of the vaccine’s 3D structure

The Galaxy Web server completed a refining procedure to enhance the structure's quality after selecting the best 3D model. As a consequence, five improved models were created by the server. When comparing two protein structures, the Global Distance Test—High Accuracy (GDT-HA) score determines how similar they are. Calculating the distance between atoms is done using the Root Mean Square Deviation (RMSD) score. A score in the allowed range for RMSD is typically between 0 and 1.2. A lower RMSD value indicates better stability. When the MolProbity score is lower than the initial model, it means that the 3D model's crucial mistakes have been reduced. The number of unfavourable all-atom steric overlaps is reflected in the clash score, and the refinement should lower the clash score. The size of energetically preferred areas is represented by the Ramachandran plot score, and often a number higher than the starting model is desirable. Based on the specifications, Model 2 was selected see Fig. 6B. Model 2's GDT-HA score of 0.8826 indicates similarity with the original 3D model. The model is the most stable, according to the low RMSD for the atomic distance score of 0.623. The MolProbity 2.385 is less than the original value, which suggests that critical mistakes have decreased. Clash scored 14.5, Poor Rotamers 0.5, and Rama scored 81.3 indicating that this model is favoured and can be used for further analysis.

### Validation of 3D structure

**Figure 7:**
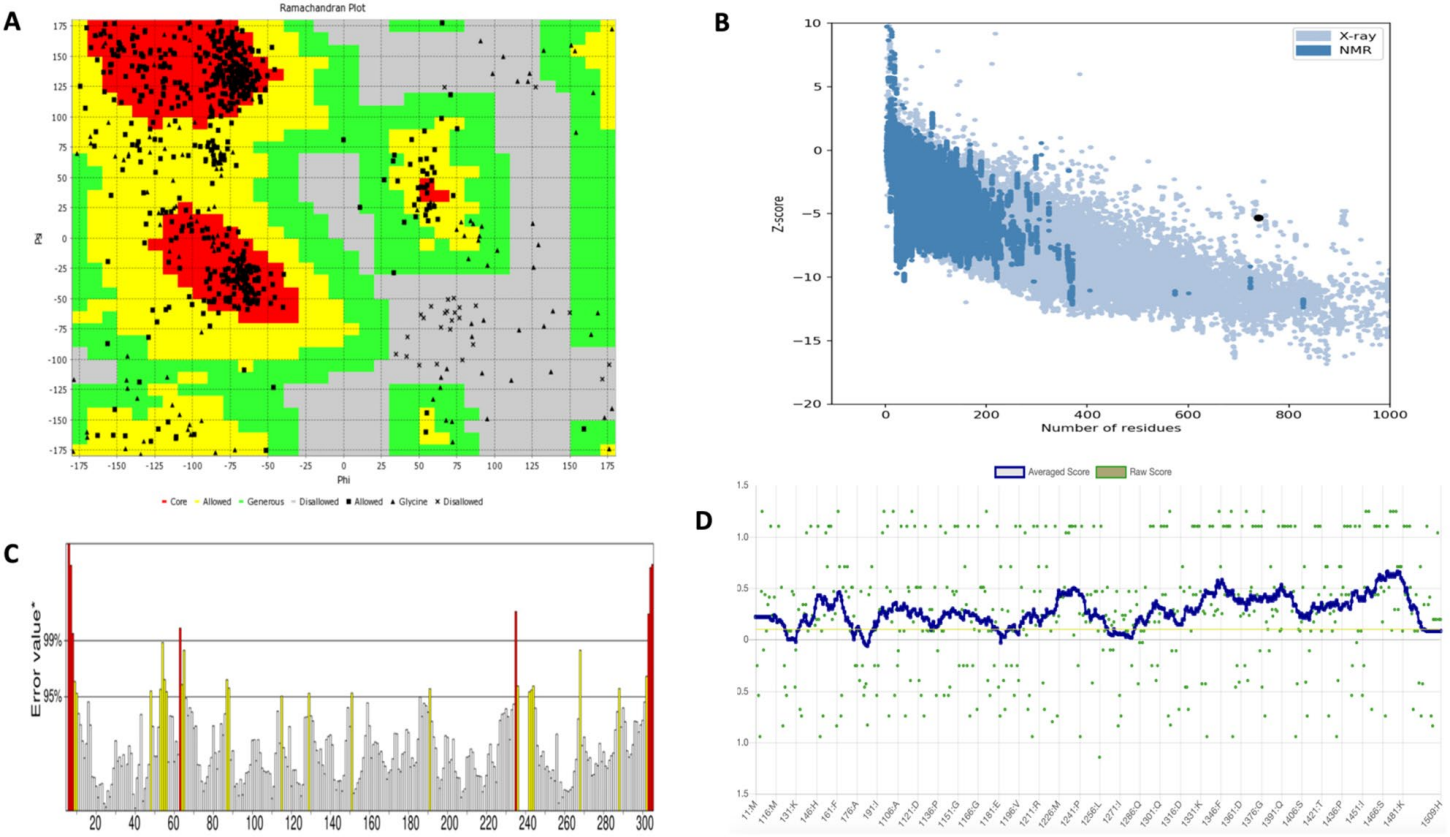
3D structure validation (**A**) When the modified model was validated using a Ramachandran plot, the findings showed the presence of 72.0%, 21.6%%, and 4.9% of the protein residues in preferred, allowed, and outlier regions. (**B**) This analysis's Z-score from ProSA-web was − 5.32. (**C**) ERRAT validated the vaccine structure with a score of 76.3158. (**D**) Verify 3D gave a score of 87.43% which signifies a good 3D structure.

Among the five refined models that were produced by the Galaxy Refine server, model 2 was subjected to further analysis having the best structure. Ramachandran plot analysis was obtained from the VADAR server (Fig. 7A). As estimated by the PROCHECK server 72.0% of residues were in the most favoured region, 21.6% were in the region additional allowed and 1.5% were in the region with general allowed. There are very few residues 4.9% in the region that are disallowed. In other to perform more validation for the refined model, the mistakes in the 3D model's quality and likelihood of inaccuracy were checked using ProSA-web. The chosen model has an overall Z-score of -5.32 with ProSA-web following refining (Fig. 7B). Furthermore, ERRAT validated the vaccine structure with a score of 76.3158 as shown in Fig. 7C representing a good structure since the score is higher than 50% but also less than 95% which is the rejection limit. Finally, VERIFY 3D in Fig. 7D reports that 87.43% of the residues in the projected model have an average 3D-1D score of > 0.1, indicating a stable structure. Different model validation revealed that the refined model had excellent quality and great stability. Therefore, this model after being analysed by VADAR, PROCHECK, and ProSA-web, ERRAT and VERIFY 3D was utilized for further analysis.

### Molecular docking between receptor and refined vaccine

**Figure 8:**
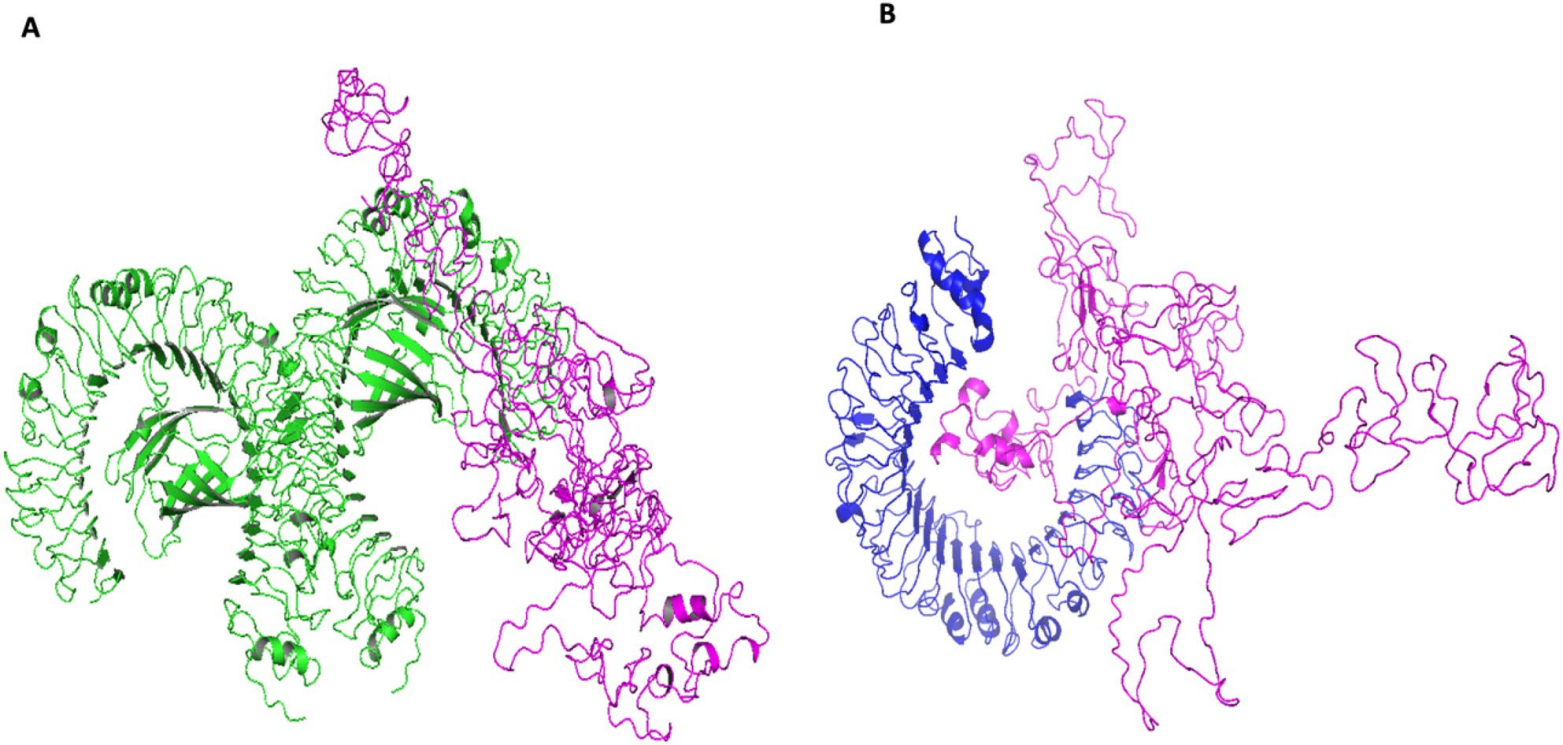
Interaction between Receptors and the vaccine (**A**) TLR3 receptor is presented in green while the vaccine is in magenta (**B**) TLR4 is presented in blue and also vaccine in magenta. This shows good interactions between the vaccine and all the receptors.

Under the cluster scores, Cluspro forecasted 22 and 30 docked models for the TLR3 as well as TLR4 vaccine receptor complexes, respectively. Among the top 10, model 7 in the TLR3 (Fig. 8A) complex having the lowest energy score of − 982.6 was chosen. Similarly, model 9 of the TLR4 (Fig. 8B) complex which had the lowest energy score of − 923.8 was chosen. These models were chosen as the best-docked complexes respectively. This denotes the possibility of molecular interaction between the anticipated vaccine design and TLR3 and TLR4 receptors.

### Codon optimisation

**Figure 9:**
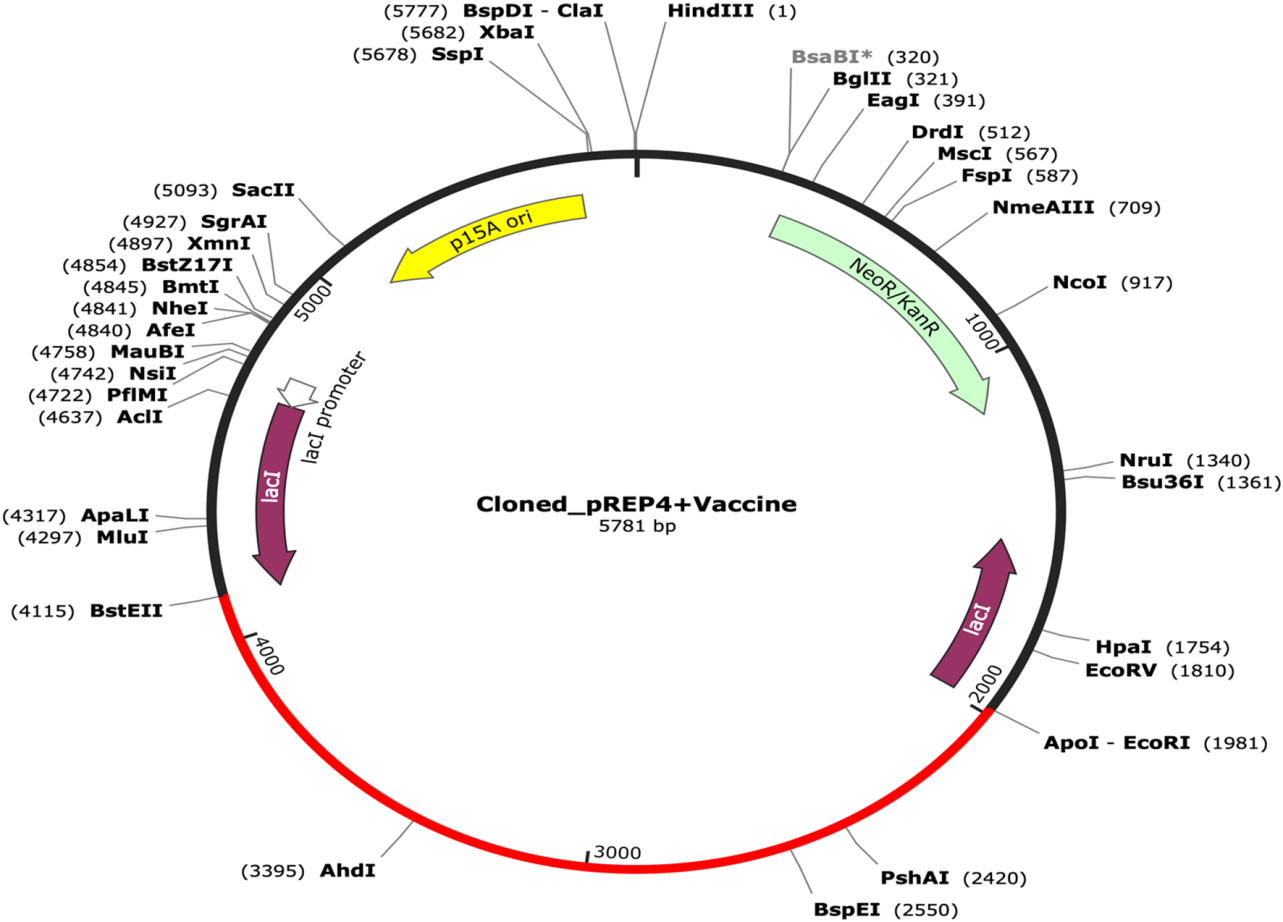
The vaccine's sequence has been optimized and put between ApoI (1981) and BstEII (4115) in the plasmid vector pREP4. The vector is black, whereas the red colour denotes the inserted DNA sequence.

The expression of recombinant proteins was improved by using a codon optimization strategy. To calculate the levels of protein expression, the codon adaptation index (CAI) values and GC contents of the E. coli (strain K12) codon system were obtained using the Java Codon Adaptation Tool (JCat) service. The optimized codon sequence has a length of 2217 nucleotides. The modified sequence had an average GC content of 61.29%, and the expected codon optimization index (CAI) value of 1.0 suggests that the E. coli would express it strongly. Finally, the recombinant plasmid sequence was produced by inserting the codon sequences into the plasmid vector pREP4 see Fig. 9 using the SnapGene programme.

### Immune simulation

**Figure 10:**
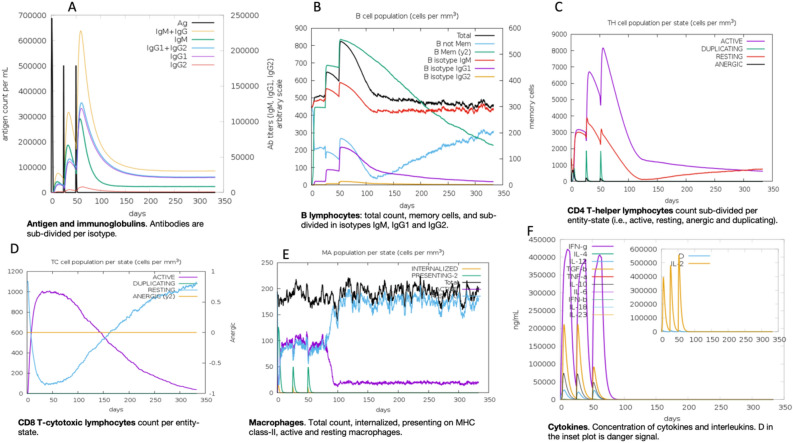
Predicting the immune response to the vaccine design using C-ImmSim simulation webserver. In response to the administration of three doses of the vaccine design both primary and secondary immune reactions were produced with a reduction in the amount of antigen.

The ImmSim server's immune simulation revealed similarities with real-life responses. Increased IgM levels showed a prompt initial response (Fig. 10A). A reduction in the concentration of the antigen was also directly correlated with immunoglobulin expression (IgG + IgM), (IgG1 + IgG2, and IgM), as well as B cell population expansion (Fig. 10A,B). TH and TC cells dramatically increased as memory evolved (Fig. 10C,D). Additionally, the initial immunization caused both the number of active and dormant macrophages to change simultaneously. After the second and third vaccinations (Fig. 10E), resting macrophages grew quickly whereas active macrophages rapidly declined. Following vaccination, it was shown that IFN- production was also enhanced (Fig. 10F). It should also be highlighted that the immunological factors IFN- and IL-2, which are essential for the immune response, are high. The results of the immune simulations indicated that administering the vaccine in three shots was sufficient to generate different immunoglobulins. A rise in IgM levels indicated the primary response, whereas increases in IgM + IgG, IgG1 + IgG2, IgG1, IgG2, and B-cell populations indicated the secondary response. There was a reduction in the number of antigens after three injections of the vaccination. The immunogenicity of T cell epitopes in the vaccine design was shown by an increase in response from both CTL and HTL T cell populations^86^. These findings showed that vaccination can effectively trigger both innate and adaptive immune responses, making it a promising candidate for a vaccine.

## Discussion

Tuberculosis (TB), is a disease that poses a danger to human life worldwide, since the BCG vaccination only provides transient protection against it, new candidate vaccines are urgently needed. In this study, a deep learning framework based on DCNN and Bi-LSTM was combined to form a model for the prediction of MtbMEV subunits. The DCNN-BiLSTM model was compared to different machine learning models. The DCNN-LSTM model consistently exceeds other methods on different sizes of datasets, showing its accuracy and success in discovering TB epitopes, although the RF and DT versions also worked well. First, H37Rv linear B-cell and T-cell epitopes containing both MHC-1 and 2 were collected from the IEDB database to build the model. The data consists of 8 million epitopes that were used to train the deep learning model. There are very limited deep learning tools for the prediction of epitopes, and to the best of our knowledge, this is the first deep learning model for the design of a TB vaccine. The trained model was used to predict MtbMEV subunits against six proteins of Mtb H37Rv; Rv1198, Rv2519, Rv3621c, Rv3344c, Rv0050, Rv3810 obtained from the mycobrowser database. Fifteen subunits were predicted as possible MtbMEV from these proteins. With this model, it is possible to design a MEV within minutes against TB.

To confirm, the suitability of the 15 subunits predicted by the proposed model for vaccine design, different evaluations were performed. The toxicity and allergenicity were ruled out and the antigenicity was confirmed. It was seen that all the subunits were non-toxic although four subunits were not antigenic or had allergic property, and were therefore excluded from the vaccine. Next, the subunits were subjected to B-cell, HTL, and CTL epitope predictions using different tools. The aim of HTL and CTL epitope prediction when creating multi-epitope-based vaccines is to identify the short peptide sequence present in an antigen that elicits CD4 + or CD8 + T cell stimulation in vivo^15^. It was observed that they have good epitopes that are necessary for a vaccine. Using NetMHCpan-4.1 and NetMHCIIpan-4.0 it was found that the MtbMEV subunits consist of 108 CTL epitopes and 40 HTL epitopes predicted as strong binders. Since T-cell epitopes with MHC molecules are required for the activation of the adaptive immune system, choosing epitopes coupled with MHC is a crucial component in predicting effective T-cell epitopes. Finally, the MtbMEV vaccine consists of 11 B cell subunits, 3 HTL subunits and 7 CTL epitope subunits.

As the vaccine is processed by the cells, linkers are crucial for controlling junctional immunogenicity and maintaining the uniqueness of each epitope, guaranteeing the immunogenicity of each epitope^87^. Also, the stability of the three-dimensional structure is determined by the optimal placement of the linkers in the MEV sequence. In this work, we connected anticipated B and T-cell vaccine subunits predicted by the DCNN-LSTM using the linkers AAY and GPGPG. Also, for the highest expression and bioactivity enhancement of the vaccine, the EAAAK linker was additionally fused between the adjuvant and the epitope sequences^67^. EAAAK is a rigid peptide linker that forms α-helices and has a strong packed backbone due to intramolecular hydrogen bonding. EAAAK linkers provide effective functional domain separation by maintaining a constant distance between the epitopes with little interference, preserving each one's unique functional characteristics^88^. GPGPG is a glycine-rich linker that gives neighbouring domains great accessibility and flexibility, in addition to enhancing construct solubility^89^, also GPGPG possesses the capacity to elicit HTL immunological response as well as antigen presentation^90^. The AAY linker boosts the multi-epitope vaccine's immunogenicity. In mammalian cells, the AAY linker serves as the proteasomes' cleavage point. As a result, epitopes linked by the AAY linker efficiently divide inside the cells, improving expression and lower junctional immunogenicity^87^. MtbMEV consists of an adjuvant (Griselimycin) added to the N-terminal end to boot immune response. Adjuvants are crucial for boosting the humoral and/or cell-mediated immune response to vaccine antigens, which in turn increases the efficacy of the vaccine. As a result, creating vaccines with the right adjuvants is a desirable strategy for providing people with long-lasting, protective immunity^91^. Finally, six histidine residues were added to the C-terminal end of the vaccine.

This MEV has good antigenicity, and physiochemical properties having 67,228.96 Da molecular weight. The molecular weight of the vaccine’s protein needs to be less than 110,000 Da^92^. The MtbMEV is thermostable, soluble, and hydrophilic with good solvent accessibility. Most importantly, the possibility of an autoimmune reaction was ruled out by performing a BLAST search which reveals that the vaccine does not have a resemblance with any human protein. The secondary structure analysis revealed that it contains 87% coil, 10% beta, and 2% helix. The tertiary structure was highly upgraded after refinement, as presented by the 3D structure validation analysis. The findings of the Ramachandran plot analysis, which was used to verify the modified model, showed that 72.0%, 21.6%%, and 4.9% of the protein residues, respectively, were in preferred, allowed, and outlier sections. The MtbMEV's tertiary structure demonstrated a high number of β-turns and random coils, in line with the outcomes that the secondary structure anticipated. This suggests that the MEV has an effective antigen potential. The likelihood of the protein forming antigenic epitopes is indicated by the high percentage of β-turn and random coil seen in the MEV^93^. To study the interactions of the vaccine with the receptor, molecular docking using two receptors TLR3 and TLR4 was performed and the results demonstrate high binding towards the receptors. This signifies that there will be high immune reactions as a result of these interactions. The IMMSIM simulation confirmed the generation of innate and adaptive responses. The majority of IgM antibodies are frequently produced during the first immune response, while some IgG antibodies are also produced. Elevated IgM and IgG levels are indicative of the secondary immune response, which is triggered by a second and subsequent exposure to the same antigen. It was demonstrated that three injections were adequate to elicit a potent immunogenic response. Additionally, in-silico cloning revealed that the vaccine will be highly expressed in *E. coli*. To produce recombinant proteins, the MEV must be expressed in an appropriate E. coli expression vector^94^.

The results of this study can be further experimentally verified utilizing a variety of analyses to establish a candidate vaccine for future clinical trials. Although the deep learning models presented in this work achieved an outstanding performance, further work will be carried out with different model architectures that could be deployed as a web tool for B and T-cell epitope predictions.

## Data Availability

The datasets used and/or analyzed during the current study are available from the corresponding author upon reasonable request**.**
